# Sense of Belonging, Religious Activity, and Well-Being in Long-Term Care Residents

**DOI:** 10.1093/geroni/igaa057.1241

**Published:** 2020-12-16

**Authors:** Kelly Shryock, Suzanne Meeks

**Affiliations:** University of Louisville, Louisville, Kentucky, United States

## Abstract

Residence in a long-term care (LTC) facility poses numerous challenges to psychological well-being and rates of depression are high. Sense of belonging (SoB) has been linked with measures of well-being in all age groups and interventions focused on improving SoB have been successful with college-age adults. It is unclear if SoB improves in LTC residents as they adjust to living in this environment or what factors predict poor SoB in this population. As part of a larger study of care preferences in LTC residents, participants (n= 76) completed measures of SoB, well-being, religious activity, and demographic information. SoB did not vary significantly based on duration of stay, age, gender, ethnicity, marital status, number of children, education, facility, cognitive functioning, or physical health. SoB was found to be significantly and positively correlated with participation in religious activities (r= .388, N=76, p=.001), private religious practices (r= .275, N=71, p=.020), and spirituality (r= .263, N=70, p=.028). There was also a significant positive correlation between SoB and positive affect (r= .450, N=74, p<.001) and SoB and life satisfaction (r= .393, N=74, p=.001). These results suggest that connections formed before admission to a LTC facility, including religious networks, are important to SoB and well-being and that individuals without or with low religious involvement may benefit most from interventions focusing on improving SoB in LTC residents.

